# The protein-protein interaction network of eyestalk, Y-organ and hepatopancreas in Chinese mitten crab *Eriocheir sinensis*

**DOI:** 10.1186/1752-0509-8-39

**Published:** 2014-03-27

**Authors:** Tong Hao, Zheng Zeng, Bin Wang, Yichen Zhang, Yichen Liu, Xuyun Geng, Jinsheng Sun

**Affiliations:** 1 Tianjin Key Laboratory of Animal and Plant Resistance/College of Life Science, Tianjin Normal University, Tianjin 300387, P.R. China; 2 Tianjin Aquatic Animal Infectious Disease Control and Prevention Center, Tianjin 300221, P.R. China

**Keywords:** *Eriocheir sinensis*, Chinese mitten crab, Network integration, Signal transduction, Evolution path

## Abstract

**Background:**

The protein-protein interaction network (PIN) is an effective information tool for understanding the complex biological processes inside the cell and solving many biological problems such as signaling pathway identification and prediction of protein functions. *Eriocheir sinensis* is a highly-commercial aquaculture species with an unclear proteome background which hinders the construction and development of PIN for *E. sinensis*. However, in recent years, the development of next-generation deep-sequencing techniques makes it possible to get high throughput data of *E. sinensis* tanscriptome and subsequently obtain a systematic overview of the protein-protein interaction system.

**Results:**

In this work we sequenced the transcriptional RNA of eyestalk, Y-organ and hepatopancreas in *E. sinensis* and generated a PIN of *E. sinensis* which included 3,223 proteins and 35,787 interactions. Each protein-protein interaction in the network was scored according to the homology and genetic relationship. The signaling sub-network, representing the signal transduction pathways in *E. sinensis*, was extracted from the global network, which depicted a global view of the signaling systems in *E. sinensis*. Seven basic signal transduction pathways were identified in *E. sinensis*. By investigating the evolution paths of the seven pathways, we found that these pathways got mature in different evolutionary stages. Moreover, the functions of unclassified proteins and unigenes in the PIN of *E. sinensis* were predicted. Specifically, the functions of 549 unclassified proteins related to 864 unclassified unigenes were assigned, which respectively covered 76% and 73% of all the unclassified proteins and unigenes in the network.

**Conclusions:**

The PIN generated in this work is the first large-scale PIN of aquatic crustacean, thereby providing a paradigmatic blueprint of the aquatic crustacean interactome. Signaling sub-network extracted from the global PIN depicts the interaction of different signaling proteins and the evolutionary paths of the identified signal transduction pathways. Furthermore, the function assignment of unclassified proteins based on the PIN offers a new reference in protein function exploration. More importantly, the construction of the *E. sinensis* PIN provides necessary experience for the exploration of PINs in other aquatic crustacean species.

## Background

The development of high throughput techniques supplies a rich source of information for the Protein-protein Interaction Network (PIN) research. The interpretation of such information is a key to understand the complex world of biological processes inside the cell [1]. Knowledge of PINs helps researchers to solve many problems such as signaling pathways identification [2], recognition of functional modules [3] and prediction of protein functions [4]. Given the significant importance of PINs, proteome-wide interaction networks based on protein interactions has been constructed for many organisms [5-7]. The early study of PIN mostly focused on *Saccharomyces cerevisiae*. Schwikowski et al. performed a global analysis of published proteins interactions in *S. cerevisiae* and predicted the functions of 364 previously uncharacterized proteins [8]. Some interesting sub-networks were extracted from the PINs of *S. cerevisiae* and analyzed, for example, the spindle pole body related sub-network in Ito T’s work [9] and DNA damage response data set in Ho Y’s work [10]. Construction and analysis of PINs for other microorganisms has been subsequently performed, such as the PINs of *Drosophila melanogaster *[11], *Helicobacter pylori *[12] and *Bacillus subtilis *[13]. In the decades-long development of PIN, interest has shifted from microbial systems [14,15] to mammalian [16] and more kinds of organisms [5]. However, to date, there is no large-scale PIN available for the study of aquatic crustacean. Although much effort has been made on the phenotype or physiological study of aquatic animals and crustaceans [17], an important ongoing problem is that the original inducement of all the phenotype and physiological features is the expression of genes and interaction of proteins. However, the expression and interaction of genes and proteins are still indistinct in most aquatic animals. As the protein interactions based on the gene expression has a significant role in the in-depth exploration of the biological process mechanism in cells, a PIN is necessary and important for the systematic study of aquatic crustaceans.

The Chinese mitten crab (*Eriocheir sinensis*) (Henri Milne Edwards, 1854) is one of the most important aquaculture species in China with high commercial value as a food source [18]. Many studies have been performed focusing on single or several genes [19], proteins [20] or a specific pathway [21] to accelerate the growth or improve the immune and signal transduction system of *E. sinensis*. However, the genome sequence of any *E. sinensis* species is still unavailable. Therefore, a whole map of the protein interactions in *E. sinensis* is still fragmentary and different signaling pathways implicated in growth and immune response also remain incomplete. Recently, Illumina RNA-seq, the next-generation deep-sequencing technique, provides new approaches to obtain a whole transcriptome sequencing [22,23], which makes it possible to get huge amounts of knowledge on *E. sinensis* proteins and subsequently obtain a systematic overview of the protein-protein interaction system.

In this work we sequenced the transcriptional RNA sequences in the eyestalk, Y-organ and hepatopancreas of *E. sinensis* and presented a substantial resource of affinity-tagged proteins. A PIN of *E. sinensis* was generated based on the transcriptome sequencing. The network covers hundreds of previously-uncharacterized proteins, thus providing functional associations and biological context for the proteins that previously lacked annotation. The signaling sub-network was extracted from the global PIN and the evolution paths of known signaling pathways were examined, which represents a new global view of the signaling systems in *E. sinensis*. Functional assignment of the unclassified proteins and unigenes supplies significant guidance for the *in vivo* investigation of proteins/genes related to specific function. To our knowledge, the PIN of *E. sinensis* is the first large-scale aquatic crustacean protein interaction network, thereby providing a systems biology view of an aquatic crustacean proteome.

## Results and discussion

### Transcriptome sequencing of *E. sinensis*

To obtain the *E. sinensis* transcriptome data, RNA from eyestalks, Y-organs and hepatopancreas mixed samples of *E. sinensis* were sequenced with the Illumina HiSeqTM2000. In total 2,358,728,280 nt clean nucleotides were found with Q20 and GC percentages of 96.68% and 45.08%, respectively. 26,208,092 clean reads were then obtained. From these clean reads, 157,168 contigs (mean length 236 nt) were assembled and then 58,582 unigenes (mean length 459 nt) were constructed from contigs with SOAP *de novo*, including 57,060 distinct singletons and 1,522 distinct clusters. The sequenced unigenes were subsequently aligned against the Nr database using BLASTn and BLASTx searching with E-value < 1*E-5. Finally 21,678 unigenes (37.00%) were matched. With Nr annotation, GO annotations of unigenes were obtained with the Blast2GO program. Among the total 58,582 unigenes of *E. sinensis*, 6,883 unigenes (11.75%) were annotated to the GO database with confident matches, including 4,680 assigned to the biological process category, 4241 assigned to the cellular component category and 5,684 assigned to the molecular function category. After the GO annotation of each unigene, WEGO software was used to obtain the GO functional classification for all unigenes in biological process category and to understand the distribution of gene functions from the macro level. In the biological process category, unigenes were divided into 26 different biological processes. Cellular process (3191; 68.2%) and metabolic process (2492; 53.2%) were most highly represented among them, other processed such as biological regulation (1392; 29.7%), developmental process (1094; 23.4%), localization (1166; 24.9%), multicellular organismal process (1170; 25%), regulation of biological process (1228; 26.2%) and response to stimulus (1057; 22.6%) were also included in biological process. The transcriptome sequencing and GO annotation results can be found in Additional file 1.

### The protein information of model organisms

The protein sequence data of six model organisms (*Drosophila melanogaster*, *Caenorhabditis elegans*, *Homo sapiens*, *Rattus norvegicus, Mus musculus*, *Saccharomyces cerevisiae*) were downloaded from the Uniprot database. The number of protein sequences in each organism is shown in Table 1.

**Table 1:** Number of protein sequences in model organisms from Uniprot

**Model organism**	**Protein sequences**
*D. melanogaster*	18768
*C. elegans*	24319
*H. sapiens*	81470
*R. norvegicus*	37104
*M. musculus*	54232
*S. cerevisiae*	6649

The protein interaction information of the above six model organisms was obtained from the PINA database. Usually, protein names were used in the protein interactions for presenting proteins, whereas for some interactions, the proteins in different model organisms were presented in different ways, which needed to be normalized for the convenience of the following analysis. Two special situations between protein IDs and protein names should be pretreated. Firstly, when there is no related protein name for a protein, the protein ID was used to mark the protein instead of a protein name. Secondly, sometimes the same protein is represented in different ways in different interactions. Some are marked with the protein name and the others are marked with the protein ID. In this situation, the protein name was used instead of the protein ID. For example, Vha36-2 is a protein in *D. melanogaster*. In the interaction pair: uniprotkb:Q7PLP5-uniprotkb:A1Z8V7, it is represented with the protein ID A1Z8V7 and in another interaction pair: uniprotkb:RpL15- uniprotkb:Vha36-2, it is represented with its protein name Vha36-2. We replaced A1Z8V7 with Vha36-2 in the first interaction to represent the protein. In this way, 44, 140, 679, 11, 10 and 40 protein names were pretreated in *C. elegans*, *D. melanogaster*, *H. sapiens*, *M. musculus*, *R. norvegicus* and *S. cerevisiae*, respectively. The number of proteins and protein-protein interactions of the six model organisms from PINA are shown in Table 2.

**Table 2:** The protein-protein interactions from PINA

**Organism**	**Proteins**	**Protein interaction pairs**
*D. melanogaster*	9042	33006
*C. elegans*	4452	8335
*H. sapiens*	12587	72157
*R. norvegicus*	1191	1405
*M. musculus*	3242	5436
*S. cerevisiae*	6002	90199

### Construction of model-organism-based protein-protein interaction sub-network

In order to construct the PIN network of *E. sinensis*, the model-organism-based protein-protein interaction sub-networks were first constructed. The sub-networks were constructed based on the sequence alignment of model organisms and *E. sinensis*. According to the alignment result, the homologous sequence existing in each protein interaction of a model organism was marked. If two proteins in an interaction can both be matched with the unigenes in *E. sinensis*, then these two proteins and their interaction were considered as part of the model-organism-based sub-network. In this way, 6 different protein-protein interaction sub-networks were constructed based on the protein information of 6 model organisms respectively. The numbers of proteins, related unigenes and interactions in each model-organism-based protein-protein interaction sub-network are shown in Table 3. The unigenes and proteins in the sub-networks are not in one-to-one relationships. For example, protein yrt in *D. melanogaste* based sub-network is related to two unigenes (Unigene23137_A0A and Unigene37966_A0A) in *E. sinensis*.

**Table 3:** **Features of protein-protein interaction sub-networks of *E. sinensis***

**Organism**	**Protein-protein interaction sub-network**		
	**Unigene**^**a**^	**Protein**^**b**^	**Protein-protein interaction pair**^**c**^
*D. melanogaster*	5269(9.00%)	2637(27.52%)	7968(19.20%)
*C. elegans*	1515(2.59%)	792(16.15%)	912(8.99%)
*H. sapiens*	3017(5.15%)	1976(13.14%)	8170(7.66%)
*R. norvegicus*	668(1.14%)	351(20.73%)	364(16.07%)
*M. musculus*	878(1.50%)	489(11.79%)	543(6.73%)
*S. cerevisiae*	3393(5.79%)	1468(23.93%)	22675(20.14%)

^a^The number in bracket shows the proportion of related unigenes accounting for all the unigenes in the model organism.

^b^The number in brackets shows the proportion of proteins in the sub-network accounting for all the proteins in the model organism.

^c^The number in brackets shows the proportion of interaction pairs in the sub-network accounting for all the interaction pairs in the model organism.

### Construction of PIN for *E. sinensis*

The PIN of *E. sinensis* was constructed according to the method described in the Methods section. The organism with a closer relationship to *E. sinensis* was integrated preferentially. *D. melanogaster* belongs to the Arthropoda-Insecta, which has the closest genetic relationship with *E. sinensis* (Arthropoda-Crustacea) compared with the other 5 organisms. *C. elegans* belongs to Protocoelomata-Nematomorpha, which is more primitive than Arthropoda-Crustacea, but has a closer genetic relationship with *E. sinensis* than the other 4 organisms. Therefore, *D. melanogaster* and *C. elegans* based sub-networks were used as the target and query networks respectively in the first turn integration. Subsequently the *H. sapiens*, *R. norvegicus*, *M. musculus* and *S. cerevisiae* based sub-network were aligned and integrated in order. One thing to mention is that *R. norvegicus* and *M. musculus* actually have similar genetic relationship with *H. sapiens*. However, the protein and interaction information of *H. sapiens* from Uniprot and PINA database is much more than that of *R. norvegicus* and *M. musculus*. Considering the attribution of the organisms to the PIN reconstruction, the *H. sapiens* based sub-network was integrated prior to *R. norvegicus* and *M. musculus*. Various numbers of protein interactions were added to the integrated network in each turn of integration and thus the PIN of *E. sinensis* was expanded based on the model organisms. Finally the PIN of *E. sinensis* was obtained after five-turn integrations of the above 6 sub-networks. The final *E. sinensis* PIN contained 3,223 proteins and 35,787 interaction pairs (Additional file 2). The scale of each intermediate integrated network after each turn of integration is shown in Table 4.

**Table 4:** The scale of integrated network after each turn of integration

	**First turn**	**Second turn**	**Third turn**	**Fourth turn**	**Fifth turn**
Node	2756	3058	3076	3096	3223
Edge	8784	16049	16324	16670	35787

The PIN of *E. sinensis* is composed of a largest weakly connected component (LWCC) and several small components. As the LWCC contains most of the nodes and edges in the global PIN, the topological features of the LWCC were analyzed and compared with that of the model-organism-based sub-networks. The basic features of LWCCs are shown in Table 5. Actually, the features of LWCCs from different sub-networks are quite different from each other. For example, the number of nodes and edges in *D. melanogaster* based sub-network is much more than that in *C. elegans* based sub-network, and the diameter is smaller, which means the connectivity of the *D. melanogaster* based sub-network is stronger than that of the *C. elegans* based sub-network. The smaller path length further proved this conclusion. The *S. cerevisiae* based sub-network obviously has the strongest connectivity. Generally the topological parameter values of *E. sinensis* are between that of *S. cerevisiae* and the other five organisms, indicating that the PIN of *E. sinensis* assimilates the information in the six model organisms as well as eliminating the redundant information.

**Table 5:** **The topological features of the LWCC in model-organism-based sub-networks and *E. sinensis *** **PIN**

**Sub-network/network**	**Node**	**Edge**	**Diameter**	**Average path length**	**Clustering coefficent**	**Average degree**	**Index aggregation**
*D. melanogaster*	2,515	7,890	12	4.578	0.118	6.27	95.37%
*C. elegans*	661	838	16	6.318	0.024	2.54	83.46%
*H. sapiens*	1,906	8,115	10	3.55	0.161	8.52	96.46%
*R. norvegicus*	262	289	15	4.118	0.037	2.21	74.64%
*M. musculus*	314	408	15	5.442	0.086	2.6	64.21%
*S. cerevisiae*	1,464	22,672	5	2.29	0.327	30.97	99.73%
*E. sinensis*	3,196	35,769	8	3.15	0.178	22.38	99.16%

### Score of protein-protein interaction pair

**Figure 1:**
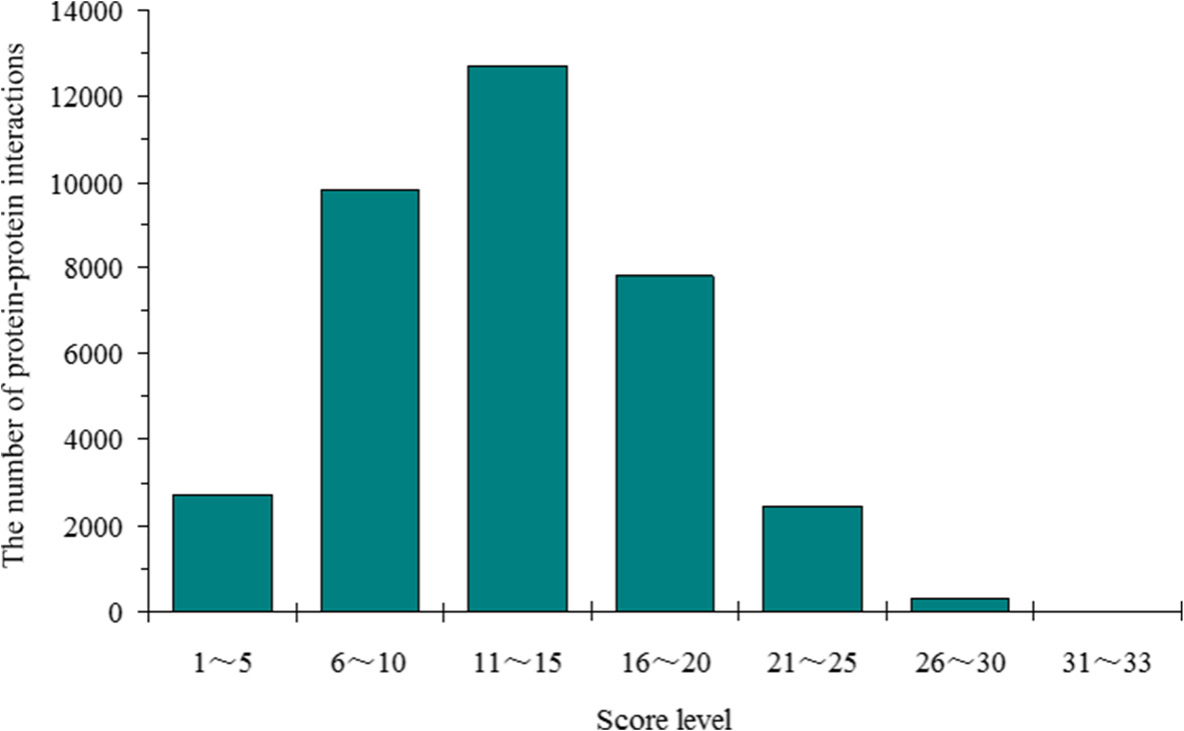
**Score distribution of *E. sinensis *** **protein-protein interaction pairs.**

As the proteins (nodes) and interactions (edges) in the PIN came from different model organisms with various genetic relationships with *E. sinensis*, in order to represent the confidence of the interaction pairs, the score of each protein-protein interaction pair was evaluated according to the method described in the Methods section. The highest score is 33, reached by 5 interaction pairs. The highest score of edges and nodes are 14 and 10, respectively. Figure 1 shows the score distribution of the interaction pairs in the PIN of *E. sinensis*. 35.35% interaction pairs distribute in scores of 11–15. The interaction pairs with scores of 6–10 and 16–20 make up 27.83% and 21.54% of all the interaction pairs respectively. The score of each interaction pair is shown in Additional file 2.

**Figure 2:**
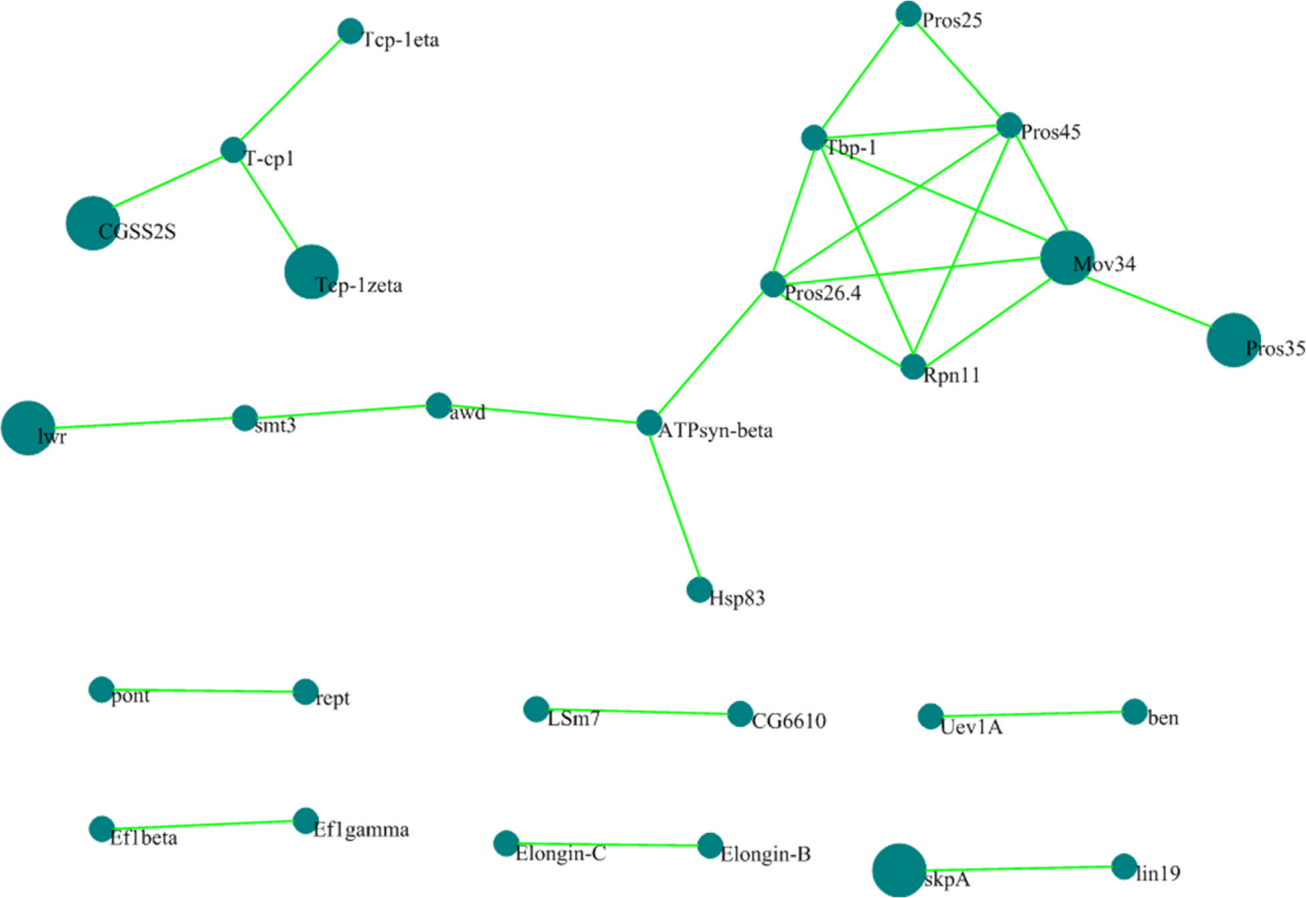
**The network with score greater than or equal to 30 in *E. sinensis *PIN.** The big circle nodes are the hub proteins with degree larger than 33.

The number of interaction pairs with scores above or equal to 30 is only 27 (Figure 2). These interactions exist in most of the model organisms, indicating their conservatism. For example, proteins Pros26.4, Mov34, Pros45, Tbp-1 and Rpn11 in these interactions are part of 26S protease. Mov34 is a regulation subunit and Rpn11 is a metal protease component of 26S protease. 26S protease is a huge protease complex widely found in many organisms [24]. Pros35, Mov34, CG5525, Tcp-1zeta, lwr and skpA are the hub proteins with their degree larger than 33, which exist in at least four model organisms and have important biological function in the metabolism by influencing many of their neighbor proteins.

### Identification of signaling sub-network in *E. sinensis*

**Figure 3:**
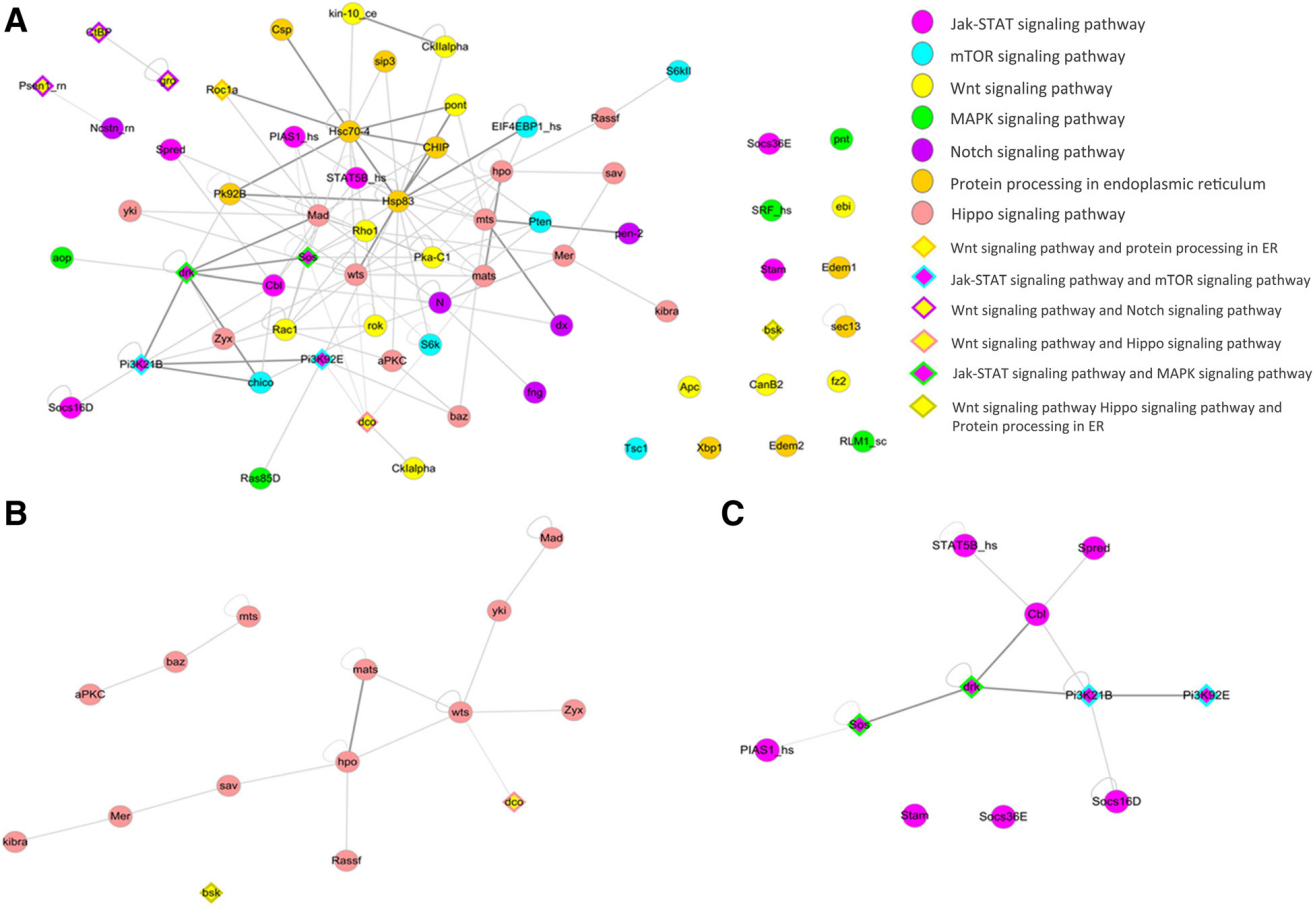
**The signaling sub-network of *E. sinensis. *A**: The 68 proteins in the 7 known pathways. **B**: Hippo signal transduction pathway. **C**: Jak-STAT signal transduction pathway.

Signaling pathways are significant mediates of cell growth, development, immune and other life activities, which play a crucial role in almost all growth stages. To better understand the signaling systems of *E. sinensis*, the proteins with GO annotation “signaling” (GO:0023052) and their related interactions were extracted from the global PIN to generate the signaling sub-network. There are in total 572 proteins with GO:0023052 in *E. sinensis*, in which 67 are isolated and 505 interacted with other protein(s), the number of protein-protein interaction relationships is 2039 (Additional file 3). Seven known signal transduction pathways in the KEGG database were found in *E. sinensis*: Hippo, Jak-STAT mTOR, Wnt, MAPK, the Notch signal transduction pathway and the protein assembly process in endoplasmic reticulum. Some proteins are active in two or more signal pathways. Proteins in the above 7 pathways also interacted with other proteins in the PIN and finally the sub-network contains 313 proteins and 1,579 interaction pairs, including the 68 proteins in known signaling pathways and their neighboring proteins. The 68 proteins with 130 interaction pairs in known signaling pathways are shown in Figure 3A, in which 14 proteins and 1 interaction pair are isolated from the fully connected sub-network.

The Hippo signal transduction pathway is responsible for the growth inhibition of cells, which is a highly conservative pathway. It was first found in *D. melanogaster* and has been found in many mammals such as *R. norvegicus* and *H. sapiens*. The Hippo signal transduction pathway has significant function in organ size control, stem cell self-renewal, cancer inhibition and tissue homeostasis in response to multiple stimuli, including cell density and mechanotransduction [17,25,26]. Proteins wts, hpo and sav in this pathway are found to be responsible for cancer inhibition. The interaction of hpo and sav is able to phosphorylate and activate the complex composed of wts and Mats [27]. Two top cell skeleton signal proteins Mer and Ex can be reciprocally activated with kibra to further activate the Hippo pathway [28]. In addition, wts can directly phosphorylate, and thus inhibit the activating transcription factor yki. And yki is closely related with cell multiplication and apoptosis [25]. The Hippo related proteins wts, hpo, sav, Mats, Mer, yki and kibra were found in the signaling sub-network of *E. sinensis* (Figure 3B), indicating that the Hippo signal transduction pathway also exists in *E. sinensis*. The growth control and cell self-renewal of *E. sinensis* is probably dominated by the Hippo pathway.

The Jak-STAT signal transduction pathway is composed of the PTK related receptor, PTK JAK and transcription factor. It is simulated by cytokine and participates in many important biological processes such as cell multiplication, differentiation, apoptosis and immunoregulation [29]. The PIAS protein in this pathway can inhibit activation of the STAT protein by blocking the binding activity of the transcription factor and DNA. In addition, it is reported that PIAS can interact with more than 60 proteins, many of which are immune-system-related [30]. The STAT and PIAS proteins were found in the signal network of *E. sinensis*,coming from the *H. sapiens* based sub-network. The other proteins in the Jak-STAT pathway came from the *D. melanogaster* based sub-network, such as the suppressor of cytokine signaling (SOCS), which inhibits the phosphorylation of STAT by combining and blocking JAK or competing for the phosphotyrosine site on the cytokine receptor with STAT (Figure 3C). The multiplication, differentiation and apoptosis of *E. sinensis* are possibly controlled by the Jak-STAT pathway. The different source of proteins indicated that the integration process provided more information for the PIN of *E. sinensis*.

In addition, the mTOR, Wnt, MAPK, Notch and protein processing in endoplasmic reticulum were also found in the signaling sub-network of *E. sinensis*. The mTOR pathway is a central regulator for both cell proliferation and cell growth [31]. The Wnt pathway is involved in virtually every aspect of embryonic development and also controls homeostatic self-renewal in a number of adult tissues. Many studies report that mutation of the Wnt pathway is closely related to several hereditary diseases and cancers [32]. The Notch pathway is first found in *D. melanogaster* and participates in the regulation of cell multiplication, differentiation, and apoptosis, and acts as an important regulator of immune cells development [33]. The seven signaling transduction pathways found in *E. sinensis* represent the regulation of basic cell life activity about growth, development, reproduction and disease-resistance. The signaling sub-network of *E. sinensis* provides substantial information of the signal transduction pathways and unknown proteins which need to be further studied.

### Evolution path of *E. sinensis* signaling network

The signaling network has been used to understand evolution in multicellular animals [34]. As the *E. sinensis* signaling sub-network was obtained from the integration of six model organisms and these organisms are located in different evolutionary stages, in order to promote understanding of the evolution of the signaling sub-network in *E. sinensis*, we examined the evolution path by comparing the *E. sinensis* signaling network with the six model organisms, and investigated the original organisms and preferred evolution paths of the *E. sinensis* signaling network. The six species were classified into three groups: primitive, bilaterian and vertebrate groups as described in Lei Li’s work [34]. The primitive group included *S. cerevisiae*. The bilaterian lineage was composed of *D. melanogaster* and *C. elegans*. All three vertebrate species were placed in the vertebrate group.

**Figure 4:**
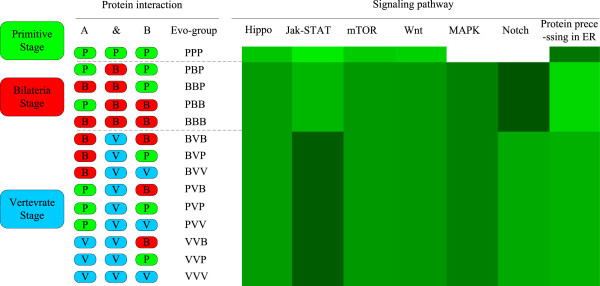
**Evolutionary origin of protein interactions in seven signaling pathways in study.** Protein interactions in the seven signaling pathways in study were divided into “Evo-groups” according to the origins of the corresponding components. A blank in the right side represents that there are no protein originating in this evo-group. For each pathway, the proportion of interactions in each evolution stage to all the interactions in this pathway is shown in different shades of green. A darker green colour stands for a larger proportion.

Based on this group partition, we identified each protein interaction in the *E. sinensis* signaling sub-network. For each protein interaction, the origin of two proteins and an interaction were defined separately using the principle in Lei Li’s work [34]. If a protein/interaction exists in a primitive organism, it is assigned to a primitive (or P) origin. If a protein/interaction exists in a bilaterian organism but not in a primitive organism, it is assigned to a bilaterian (or B) origin. If a protein/interaction exists only in vertebrate organism(s), it is assigned to a vertebrate (or V) origin. Finally, the origin of a complete protein interaction (including two proteins and an interaction) was assigned to the evolutionary stage in which the last component in the protein interaction appeared. The origin groups of seven known signaling pathways were examined (Figure 4). We found that different signaling pathways had various evolutionary paths. Protein processing in ER is relatively complete in the primitive stage with more than half of the interactions in this stage. Most interactions in the Hippo pathway exist in the bilateria and vertebrate stages, which is consistent with its first discovery in *D. melanogaster *[35]. Several interactions in the Hippo pathway originated from the primitive stage but no complete path was formed until it was found in *D. melanogaster*. The same situation also appears in the Jak-STAT, mTOR and Wnt pathway, indicating that these pathways may grow in the primitive stage and mature in the bilateria stages. MAPK and Notch have the latest evolution origin, with all interactions found in the biliteria and vertebrate stages.

### Function assignment of unclassified proteins and unigenes

**Figure 5:**
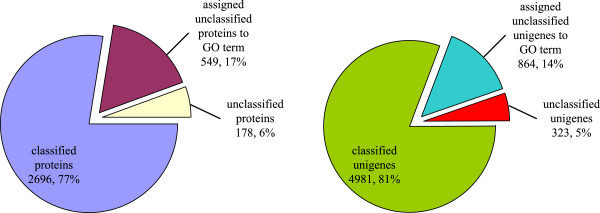
**Function distribution of unigenes in *E. sinensis.***

According to the GO biological process annotation of proteins in PIN, the functions of 2,496 proteins related to 4,981 unigenes were annotated, whereas the functions of the other 727 proteins related to 1,187 unigenes were still unknown, which makes up approximately 23% of all the proteins and 19% of all the unigenes. In order to investigate the functions of these unclassified proteins and unigenes, the method described in the Methods section was used to assign GO annotations. Finally, 549 unclassified proteins related to 864 unclassified unigenes were annotated (Figure 5), making up 76% and 73% of all the unknown proteins and unigenes (Additional file 4).

**Figure 6:**
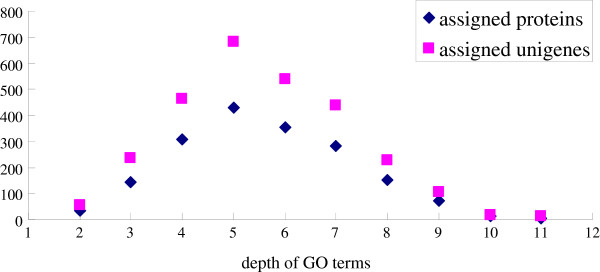
The number of classified proteins and unigenes with depth of GO term.

As the GO terms are organized in a treelike structure, we further analyzed the number distribution of newly-annotated proteins and unigenes in different GO depths (Figure 6). As GO depth increases, the annotation becomes more detailed. As shown in Figure 6, the fifth layer of the GO treelike structure contains the most assigned proteins and unigenes. As proteins or unigenes may have multiple functions, the GO annotation and proteins/unigenes are not in one-to-one relationship. 18 proteins were annotated as immune response-related proteins. They functioned in innate immune response (4 proteins), humoral immune response (2 proteins), mucosal immune response (5 proteins), regulation of innate immune response (6 proteins) and unclear immune response (7 proteins). Furthermore, 135 signaling-related proteins were found. They acted as receptors of signaling factor or the regulators of signaling pathways. For example, 16 proteins (Q8IQV6, Q7JXG9, Q8IR25, D3ZE26, Q9V3S7, Q9V3A8, Q7KMH9, Q7JPS2, Q9VVK8, Q9W1A7, Q7KN04, Q8SZY2, Q9VUP0, Q9VVU1, Q9VLK8, A1ZA45) were annotated as Hippo signaling cascade and 12 other proteins (Q8IQV6, Q7JXG9, Q8IR25, Q9V3S7, Q9V3A8, Q7KMH9, Q7JPS2, Q9VVK8, E1JII4, Q8SZY2, Q9VVU1, Q9VLK8) functioned as regulators of Hippo signaling cascade. Although the functions of the proteins/unigenes still need to be further validated, the assignment of functions provides important reference for identification of the targets in the *in vivo* experiment.

## Conclusion

With the improvement in high-throughput sequencing technology, RNA sequencing and annotation are possible for further analysis and detection in the pursuit of certain biological goals. In present work we constructed a PIN of *E. sinensis* on the basis of transcriptomics sequencing and the proteome of six model organisms. The PIN defines a primary protein interaction landscape for *E. sinensis* cells that allows study of sub-networks with specific function. Seven known pathways were identified in the signaling sub-network extracted from the global PIN. With the analysis of evolution paths for these pathways, we found their differences in evolution origin. More proteins identified as neighbors of the proteins in seven identified pathways were prepared for further confirmation. Furthermore, the function assignment of unclassified proteins offers a new reference in protein function exploration. It is the first large-scale PIN of aquatic crustaceans, thereby providing necessary experience for the exploration of PIN for other aquatic crustacean species, as well as supplying a systems biological view of an aquatic crustacean interactome.

## Methods

### Obtaining of transcriptome data

Live *E. sinensis* (35–40 g in body weight) were purchased from the Tianjin Fisheries Institute and raised in fiberglass tanks. *E. sinensis* were cultured in freshwater at 18–20 degree centigrade (photoperiod L12:D12) for 7 days to acclimate to the laboratory conditions. Then three tissues including eyestalk, Y-organ and hepatopancreas were separated and collected. All samples were immediately frozen in liquid nitrogen and were stored at minus 80 degree centigrade until use. All experimental procedures were conducted in conformity with institutional guidelines for the care and use of laboratory animals in Tianjin Fisheries Institute and conformed to the National laboratory animal management regulations (Publication No. 2, 1988) approved by the National Science and Technology Commission.

Total RNA from *E. sinensis* tissue was sequenced with the Illumina high-throughput sequencing technology by Beijing Genomics Institution (BGI). The total RNA was extracted using the TRIzol method (Invitrogen) and then equal quantities of RNA from each tissue were pooled for transcriptome analysis. The samples for transcriptome analysis were prepared using Illumina’s kit and the generated library was sequenced using Illumina HiSeq™ 2000. Then the transcriptome *de novo* assembly was carried out with the short reads assembling program-Trinity [36] to generate unigenes. GO annotation of unigenes was obtained by the Blast2GO program [37] with an E-value cut-off at 1*E^-5^. WEGO software was used to obtain the GO functional classification for all unigenes in biological process category.

### The protein sequences of model organisms

The protein sequence data of *C. elegans*, *D. melanogaster*, *H. sapiens*, *M. musculus*, *R. norvegicus* and *S. cerevisiae* was downloaded from the Uniprot database [38] (March 2012 version). The protein interactions of these model organisms were obtained from Protein-protein Interaction Network Analysis (PINA) [39,40]. PINA integrates the protein interaction information of six public databases and supplies the complete, non-redundant protein interaction information of the above six model organisms. The March 2012 version was downloaded.

### Gene ontology annotation

The Gene Ontology database [41] supplies a standardized representation of gene and gene product attributes across species and databases, including biological process, molecular function and cellular component. The gene_ontology.obo file was downloaded to obtain GO annotation from the Gene Ontology database. The GO annotation can be described as a directed acyclic graph according to the relations of GOs and a tree structure was drawn by programming. The GO numbers in each level of the tree were extracted.

### Sequence alignment

The Basic Local Alignment Search Tool (BLAST) was downloaded from the NCBI ftp platform. The BLASTX program was used to align the nucleotide sequences (unigenes) in *E. sinensis* with the protein sequences of six model organisms to construct the model-organism-based protein-protein interaction sub-networks. The nucleotide sequence is first translated into protein sequences (one nucleotide sequence can be translated into six protein sequences) and then compared with the model organism one by one. The first aligned sequence with E value below 1*E-5 was considered as the homologous sequence.

### Network integration

The construction of the PIN for *E. sinensis* is actually the integration of the 6 model-organism-based sub-networks. We developed an efficient computational procedure for integrating two PINs with reference to the global protein network alignment method in an attempt to obtain the integrated PIN [42]. The sub-networks were integrated one by one and the order was decided according to the genetic relationship of the model organisms with *E. sinensis*.

**Figure 7:**
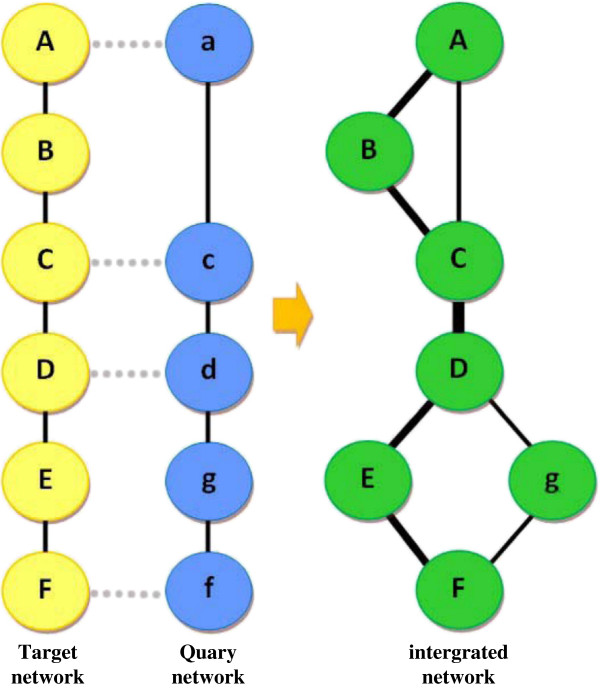
**The process of network merging.** Thickness of lines represents the score of edge. Edge with higher score has wider thickness.

When two sub-networks were prepared to be combined, firstly, the homologies of the nodes in the two sub-networks were compared. Therefore, the two sub-networks were named as the target network and the query network. All the homologous proteins in the two networks were marked and the non-homologous proteins in the query network and the protein-protein relationships uniquely existing in the query network were added into the target network to form an integrated network. The detailed processes were as follows:

(1) Proteins in the target and query networks were aligned with the BLASTP program, E value was set as 1*E-5.

(2) The first matched protein in the target network to the query network was considered to be homologous. All the homologous proteins in the two networks were extracted.

(3) The protein-protein interactions in the two networks were compared. When two proteins in an interaction pair in the query networks were both homologous to the target network (such as C-D in the target network and c-d in the query network in Figure 7), the protein names in the target network were used in the integrated network (such as C-D in Figure 7), and the new interaction pair in the query network was added if any (such as A-C in Figure 7); when only one protein in an interaction pair was homologous (such as D-E in the target network and d-g in the query network in Figure 7), then the protein name in the target network was used and the other protein in the interaction pair in the query was added (D-g in Figure 7); when no homologous proteins were found in an interaction pair in the query network, the protein names and this interaction in the query network were directly added into the integrated network.

(4) The integrated network was considered as a new target network, and another model-organism-based sub-network was used as a new query network. Then steps (1) - (3) were repeated to generate a new integrated network. Such an iterative process was stopped until all the model-organism-based sub-networks were integrated. The final integrated network was the PIN of *E. sinensis*.

### Topological features of networks

#### *Diameter and average path length of network*

In a directed network, the distance from node *i* to node *j* is the length of the shortest path between them. The diameter of a network is the length of the longest distance among all connected pairs of nodes in a graph. The average path length is the length of the distances averaged over all pairs of connected nodes in a graph [43].

#### *Connected component*

A strongly connected component (SCC) of a directed graph is a sub-graph where all nodes in the sub-graph are reachable by all other nodes in the sub-graph. Reachability between nodes is established by at least one directed path between the nodes. A weakly connected component (WCC) is a maximal group of nodes that are mutually reachable ignoring the edge directions [44].

#### *The clustering coefficient*

The clustering coefficient of a node *v* is defined as:

$$C_{v}=\frac{2e_{v}}{k_{v}\left(k_{v}‒1\right)}$$

where ***k***_***v***_ is the number of nodes in the neighbourhood of vertex *v*,and *e*_*v*_ is the number of edges existing between the neighbours of *v*. Suppose that a node *v* has *k*_*v*_ neighbours, then at most *k*_*v*_(*k*_*v*_-1)/2 edges can exist between them (this occurs when every neighbour of *v* is connected to all the other neighbours of *v*). Let *C*_*v*_ denote the fraction of these allowable edges that actually exist. The clustering coefficient of a network is defined as the average of *C*_*v*_ over all *v *[45].

#### *Degree and average degree*

In graph theory, the degree of a graph is the number of edges incident to the nodes, with loops counted twice. The average degree is the degree averaged over all the nodes in a graph [44].

#### *Index aggregation*

The Index aggregation of a network is the ratio of the nodes in the largest WCC and the global network [44].

### Score of protein-protein interaction pair

The protein interactions in *E. sinensis* PIN came from six model organisms. On one hand, proteins in different model organism were homologous to each other, as well as homologous to one or several unigenes in *E. sinensis*. On the other hand, organisms with close genetic relationship usually have similar protein interactions. Therefore, considering the homology of proteins and the genetic relationships of the model organisms with *E. sinensis*, the confidence score of the protein-protein interaction pairs in the PIN of *E. sinensis* was evaluated. Two factors were taken into consideration for scoring an interaction pair: point matching and edge matching. The point matching was actually the homology of the proteins in an interaction pair coming from different sub-networks. In the integration process, when a protein in the integrated network came from one sub-network, the score of this protein was 0. When the protein came from two homologous proteins in the target and query sub-networks, then the related unigene(s) of the two proteins were further checked. With the two proteins homologous to the same unigene(s) of *E. sinensis*, the score of their related protein in the integrated network was 2, otherwise it was 1. For example, in the first turn integration, protein CG6843 came from the integration of the homologous protein CG6843 in *D. melanogaster* and protein CIR-1 in *C. elegans*. Moreover, CG6843 and CIR-1 were both found to have high similarity with Unigene6670_A0A of *E. sinensis* by BLASTX. Therefore, the score of protein CG6843 in the integrated network was 2. Edge matching reflected the source of edge in the integrated network. The score of edges coming from the target network was higher than that from the query network because that the genetic relationship of the target organism was closer to *E. sinensis* than the query organism. Matched edge, which means the edge from both target and query network, was scored as 3. Mismatching edges from the target network and query network were scored as 2 and 1 respectively. Finally, the score of an interaction pair can be calculated as follows:

(1) $$S=\sum_{i=1}^{N}\left(\mathrm{Ai}+Ri+Bi\right)$$

where S stands for the score of a protein-protein interaction pair; A and B are the score of two nodes (proteins) in an interaction pair respectively; R is the score of the edge; i stands for the number of integration times; N (N = 5) is the maximal number of interaction times. The maximum score of an interaction pair is 35 deduced by formula (1).

### Function assignment of unclassified proteins

The functions of unknown proteins were annotated with the method mentioned in Alexei’s work [46]. The proteins were assigned to functional classes on the basis of their network of physical interactions as determined by minimizing the number of protein interactions among different functional categories [46]. Given one unknown function, we take the function that appears more often in the neighbor proteins of a known function as a prediction. Here a small change was made to Alexei’s method. In Alexei’s method, up to three of the most probable predicted functions were determined as final functions. While in the *E. sinensis* PIN, the number of function annotations for lots of known proteins is more than three. In avoid of missing important annotations, we use a parameter 25% instead of the top three rule. The detailed steps are as follows:

(1) Identify the neighbor protein(s) interacting with the protein with unknown function (unclassified). The neighbor protein(s) with GO annotation were considered as classified protein(s);

(2) Calculate the numbers of neighbor proteins with GO annotation and in the GO functional category;

(3) If the number of neighbor proteins with a certain GO functional category make up more than 25% of the total number of neighbor proteins, then the GO annotation is assigned to the unclassified protein. If only one neighbor protein with GO annotations exists, all the GO annotations were assigned to the unclassified protein;

(4) Taking into account the interactions among the above three steps, iterate (1)-(3) until no unclassified proteins can be further assigned.

## Competing interests

The authors declare that they have no competing interests.

## Authors’ contributions

TH designed the experiment and wrote the manuscript. ZZ performed the experiments. JS provided the overall project guidance, critical review and gave advice on validation of manuscript. YZ and YL contributed in the transcriptome sequencing and GO annotation. BW gave advices on the manuscript revision. XZ executed the cultivation of *E. sinensis* and supplied the tissue sample for sequencing. All authors read and approved the manuscript.

## Supplementary Material

Additional file 1 **Transcriptome sequencing and GO annotation.** Unigenes obtained from transcriptome sequencing and GO annotations. Nr annotations are also listed. GO annotations are shown in three categories: biological process, molecular function and cellular component. Click here for file

Additional file 2 **PIN of *E. sinensis.*** Scores and species source of all the proteins and interactions in *E. sinensis*. Click here for file

Additional file 3 **Signaling sub-network of *E. sinensis.*** Protein interaction pairs in signaling sub-network. Pathway information is listed in column C. Isolated nodes in the sub-network are marked in column D. Click here for file

Additional file 4 **Assigned proteins and unigenes.** Assigned proteins and related unigenes with GO annotations and GO depth information. Click here for file
